# Rising United States Hospital Admissions for Acute Bacterial Skin and Skin Structure Infections: Recent Trends and Economic Impact

**DOI:** 10.1371/journal.pone.0143276

**Published:** 2015-11-24

**Authors:** Keith S. Kaye, Dipen A. Patel, Jennifer M. Stephens, Alexandra Khachatryan, Ayush Patel, Kenneth Johnson

**Affiliations:** 1 Wayne State University and Detroit Medical Center, Detroit, Michigan, United States of America; 2 Pharmerit International, Bethesda, Maryland, United States of America; 3 Durata Therapeutics, Chicago, Illinois, United States of America; Columbia University, UNITED STATES

## Abstract

**Background:**

The number of ambulatory patients seeking treatment for skin and skin structure infections (SSSI) are increasing. The objective of this study is to determine recent trends in hospital admissions and healthcare resource utilization and identify covariates associated with hospital costs and mortality for hospitalized adult patients with a primary SSSI diagnosis in the United States.

**Methods:**

We performed a retrospective cross-sectional analysis (years 2005–2011) of data from the US Healthcare Cost and Utilization Project National Inpatient Sample. Recent trends, patient characteristics, and healthcare resource utilization for patients hospitalized with a primary SSSI diagnosis were evaluated. Descriptive and bivariate analyses were conducted to assess patient and hospital characteristics.

**Results:**

A total of 1.8% of hospital admissions for the years 2005 through 2011 were for adult patients with a SSSI primary diagnosis. SSSI-related hospital admissions significantly changed during the study period (*P* < .001 for trend) ranging from 1.6% (in 2005) to 2.0% (in 2011). Mean hospital length of stay (LOS) decreased from 5.4 days in the year 2005 to 5.0 days in the year 2011 (overall change, *P* < .001) with no change in hospital costs. Patients with postoperative wound infections had the longest hospital stays (adjusted mean, 5.81 days; 95% confidence interval (CI), 5.80–5.83) and highest total costs (adjusted mean, $9388; 95% CI, $9366-$9410). Year of hospital admission was strongly associated with mortality; infection type, all patient refined diagnosis related group severity of illness level, and LOS were strongly associated with hospital costs.

**Conclusions:**

Hospital admissions for adult patients in the United States with a SSSI primary diagnosis continue to increase. Decreasing hospital inpatient LOS and mortality rate may be due to improved early treatment. Future research should focus on identifying alternative treatment processes for patients with SSSI that could shift management from inpatient to outpatient treatment settings.

## Introduction

The US Food and Drug Administration (FDA) coined the term acute bacterial skin and skin structure infections (ABSSSIs) in a guidance document for industry released in the year 2013.[1] The FDA defines ABSSSIs as lesions with a minimum surface area of approximately 75 cm^2^ and includes cellulitis/erysipelas, wound infections, and major cutaneous abscesses. Gram-positive organisms cause the majority of ABSSSIs. Beta hemolytic streptococci and *Staphylococcus aureus* including methicillin-resistant *S aureus* (MRSA) are frequent causes of ABSSSIs; bacterial organisms less frequently causing ABSSSIs include other streptococci, *Enterococcus faecalis*, and gram-negative bacteria.[2, 3]

Increasing numbers of ambulatory patients are seeking treatment for skin and skin structure infections (SSSIs).[4–6] Results from an analysis of the National Hospital Ambulatory Medical Care Survey (NHAMCS) data show an increase in ambulatory SSSI visits from 1.35% in the year 1993 to 2.98% in the year 2005 (*P* < .001 for trend).[5] Results from a combined analysis of the NHAMCS and the National Ambulatory Medical Care Survey show a 50% increase in patient visits to physician offices, hospital outpatient departments, and emergency departments for SSSIs from the year 1997 through the year 2005.[4] Although the majority of patients presenting to ambulatory clinics for SSSI treatment can be treated as outpatients, a percentage of patients will require inpatient management.[4, 7]

Study results show that SSSI hospitalizations increased in the United States by 29% from the year 2000 through the year 2004.[8] In contrast, there was no increase in hospital admissions for pneumonia during this same time period. The largest increases were for patients with superficial infections such as cellulitis and abscess compared to those with deeper or healthcare-associated infections such as postoperative wound infections and vascular device related infections, for patients aged less than 65 years compared to those aged 65 to 100 years, and for patients hospitalized in urban compared to rural hospitals.

Study results specific to *S aureus* SSSIs show a 123% increase in hospitalizations in the United States from the year 2001 through the year 2009.[9] During this time period, *S aureus* SSSI incidence more than doubled from 57 infections per 100,000 people in the year 2001 to 117 infections per 100,000 people in the year 2009 (*P* < .001). The average associated cost of a *S*. *aureus* SSSI hospitalization was $11,622 per patient and total annual costs were estimated at $4.5 billion (2010 US$) in the United States for the year 2009.

Information on SSSI hospital admissions for adult patients since 2004 is not available in the published literature. Additionally, the effects of SSSI admissions on healthcare resource utilization in the United States are unknown. In this era of healthcare reform, a clear understanding of the current trends and the associated economic burden of SSSI hospitalizations is needed. Therefore, we performed a study to determine recent trends in SSSI hospital admissions, document healthcare resource utilization, and identify covariates associated with hospital costs and mortality for adult patients hospitalized for SSSI treatment in the United States.

## Methods

### Study Design and Data Source

We performed a retrospective cross-sectional database analysis using discharge data from the US Healthcare Cost and Utilization Project (HCUP) National Inpatient Sample (NIS) for the years 2005 through 2011. Patient characteristics and healthcare resource utilization were assessed, recent trends described, and covariates significantly associated with hospital costs and mortality were determined for patients hospitalized with a SSSI primary diagnosis. The HCUP NIS dataset is the largest publicly available all-payer inpatient care database and includes information on patient demographics, hospital characteristics, insurance coverage, primary and secondary procedures, admission and discharge status, healthcare charges, length of stay (LOS), and severity adjustment measures. [10] This stratified, random sample of data is obtained from approximately one-fifth of short-term, non-federally funded hospitals in the United States. Each year of data contained in the NIS provides information on approximately 8 million hospitalizations occurring in approximately 1,000 hospitals. The HCUP NIS dataset is a de-identified patient claims data, and hence an approval from institutional review board or patient consent was not required for this study.

### Patient Selection

We included data for all hospital admissions for patients 18 years of age or older with a primary diagnosis of SSSI. Hospital admissions with a primary SSSI diagnosis were identified by using selected International Classification of Diseases, Ninth Revision, Clinical Modification (ICD-9-CM) discharge codes (Table 1). Selected ICD-9-CM codes were consistent with infection types described in the FDA guidance on acute bacterial SSSIs.[1]

**Table 1:** ICD-9-CM codes used to identify patients with a primary SSSI diagnosis.

ICD-9-CM Code	Description
681.XX	Cellulitis and abscess of finger and toe
682.XX	Other cellulitis and abscess
686.XX	Other local infections of skin and subcutaneous tissue
958.3	Posttraumatic wound infection, not elsewhere classified
998.5X	Postoperative wound infection
035	Erysipelas

ICD-9-CM, International Classification of Diseases, Ninth Revision, Clinical Modification; SSSI, skin and skin structure infection

### Outcome Measures

Patient characteristics evaluated included age, gender, race, insurance status (eg, self-pay, Medicare, Medicaid, private), discharge disposition, and All Patient Refined Diagnosis Related Group (APR-DRGs) severity of illness (SOI) and risk of mortality (ROM) descriptors. Four APR-DRG SOI and ROM levels are defined, level 1 corresponds to minor, 2 to moderate, 3 to major, and 4 to extreme SOI or ROM. Levels represent categories and not scores and account for not only secondary diagnoses but also the interactions among the patient’s secondary diagnoses and their age, principal diagnosis, and the need for procedures.

Hospital characteristics evaluated included setting (urban or rural), location (Northeast, South, Midwest, or West), size, teaching status, and ownership. Healthcare resources evaluated included number of admissions, LOS, and hospital costs.

The NIS database contains total hospital charge data for each hospital discharge. The HCUP provides a separate hospital-specific cost-to-charge ratio file which was used to convert hospital charges to costs. Costs were then inflated to 2012 US dollars.

### Statistical Analyses

Descriptive univariate and bivariate analyses were conducted to assess patient and hospital characteristics as well as total healthcare costs. The mean and standard deviation were calculated for continuous variables; frequency and proportions were calculated for categorical variables. All analysis results were reported as annualized national estimates determined using the ‘SURVEY’ procedures (surveylogistic and surveyreg, where appropriate) in SAS v9.2 (SAS Institute Inc., Cary, NC) adjusting for the survey design of the NIS data and taking into consideration the NIS sample weights, stratification, and clustering variables. A two-sided *P*-value of < .05 was considered statistically significant.

Generalized linear models (GLM) with gamma distribution, and log link and logistic regression were used to assess variables associated with total healthcare costs and mortality, respectively. Least squares (LS) means (ie, covariate adjusted group means) were calculated from these models for LOS and cost, holding the covariates at their mean value or in the case of categorical variables, applying a distribution across levels consistent with the overall cohort (e.g., if 20% were male and 80% female within the cohort, the same distribution would be assumed for sex when calculating mean cost). Total costs were stratified by type of infection as well as length of inpatient stay. The LS means were adjusted for patient demographics (ie, age, sex, race, insurance status, year of admission, and discharge disposition), hospital characteristics (ie, region, location, size, and teaching status), and APR DRG SOI level.

## Results

**Fig 1:**
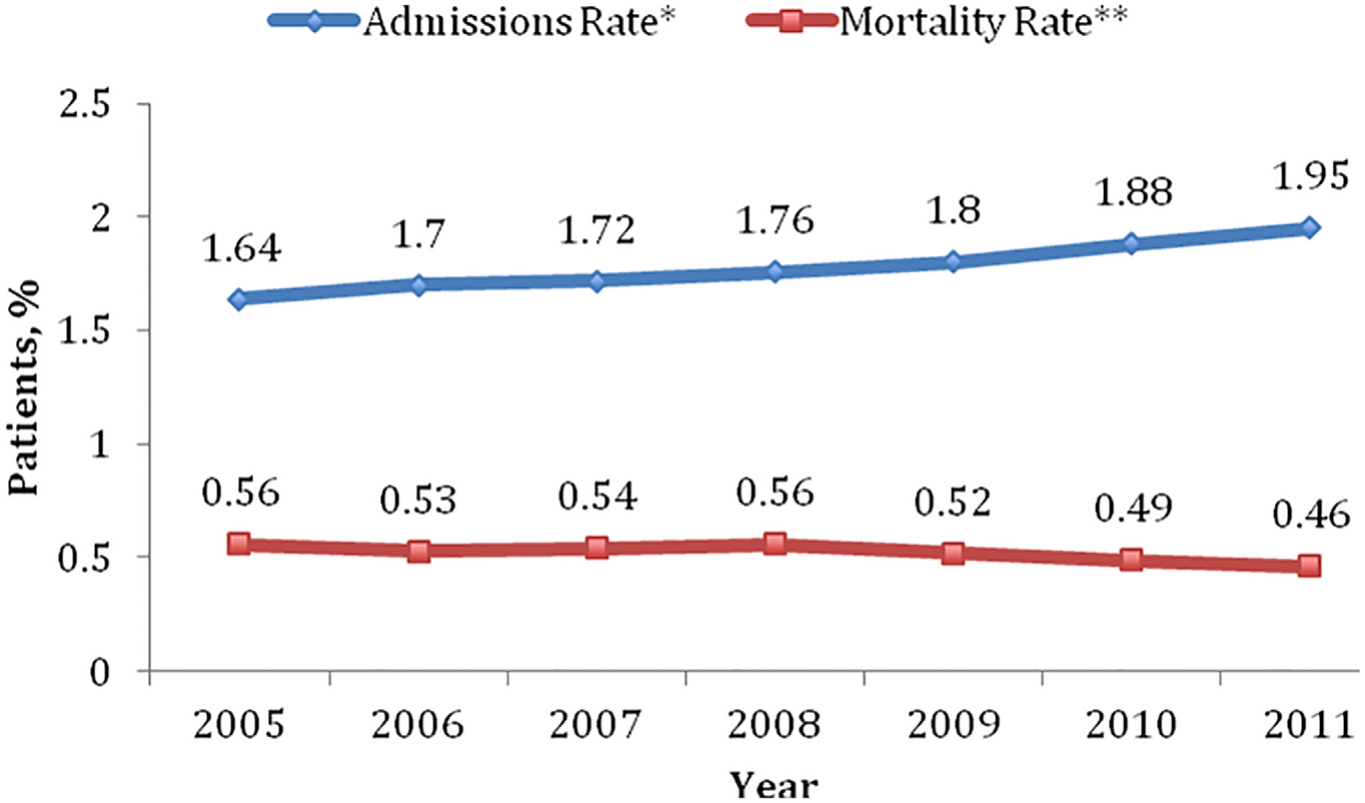
SSSI admissions by infection type, years 2005–2011. *Other infections include the following: other local infections of skin and subcutaneous tissues; posttraumatic wound infection, not elsewhere classified; and erysipelas. SSSI, skin and soft tissue infection.

Of the more than 275 million hospital admissions (weighted to national estimates) represented by the HCUP NIS for the years 2005 through 2011, a total of 4,891,187 hospital admissions (1.8%) with a primary diagnosis of SSSI were identified for patients 18 years of age or older. A total of 60.3% of patients were white, 50.8% male, 40.7% insured through Medicare, and 53.8% routinely discharged (Table 2). The most common primary SSSI diagnosis was ‘other cellulitis/abscess’ (73.5%) (Fig 1). The most common APR-DRG SOI classifications were levels I (30.1%) and II (43.5%); the most common APR-DRG ROM classification was level 1 (60.4%). Diabetes (25.2%), chronic obstructive pulmonary disease (17.3%), congestive heart failure (10.3%), moderate to severe renal disease (9.3%), peripheral vascular disease (6.2%), and diabetes with complications (5.5%) were the most prevalent comorbidities. There was a significant change in number of comorbidities over time with a significant decrease in the percent of patients without comorbidities in the 2005 to 2011 timeframe (from 3.5% in the year 2005 to 1.9% in the year 2011; *P* < .001 for the trend). In general, patient characteristics remained similar throughout the study.

**Table 2:** Patient, clinical and hospital characteristics for adult inpatients with a primary SSSI diagnosis (years 2005–2011).

Characteristics*	2005 (n = 641,863)	2006 (n = 670,819)	2007 (n = 678,876)	2008 (n = 700,803)	200 (n = 711,392)	2010 (n = 734,664)	2011 (n = 752,770)	All (N = 4,891,187)
Age, y								
18–44	200,994 (31.31)	208,437 (31.07)	204,926 (30.19)	200,231 (28.57)	199,183 (28.00)	206,706 (28.14)	204,045 (27.11)	1,424,523 (29.1)
45–64	232,300 (36.19)	247,252 (36.86)	256,089 (37.72)	263,673 (37.62)	273,980 (38.51)	284,867 (38.78)	290,979 (38.65)	1,849,140 (37.8)
≥ 65	208,568 (32.49)	215,130 (32.07)	217,861 (32.09)	236,899 (33.80)	238,229 (33.49)	243,090 (33.09)	257,746 (34.24)	1,617,523 (33.1)
Sex								
Male	324,904 (50.62)	344,219 (51.31)	347,753 (51.22)	353,922 (50.50)	360,907 (50.73)	372,575 (50.71)	378,064 (50.22)	2,482,344 (50.8)
Female	315,740 (49.19)	325,666 (48.54)	330,173 (48.64)	345,936 (49.36)	349,598 (49.14)	361,285 (49.18)	374,036 (49.69)	2,402,435 (49.1)
Race								
White	359,902 (56.07)	363,324 (54.16)	362,055 (53.33)	426,067 (60.80)	453,186 (63.70)	478,359 (65.11)	506,961 (67.35)	2,949,853 (60.3)
Black	49,525 (7.72)	61,318 (9.14)	62,633 (9.23)	60,988 (8.70)	68,154 (9.58)	83,911 (11.42)	83,274 (11.06)	469,804 (9.6)
Hispanic/Latino	50,383 (7.85)	60,029 (8.95)	54,368 (8.01)	52,418 (7.48)	60,076 (8.44)	64,528 (8.78)	67,212 (8.93)	409,014 (8.4)
Asian or Pacific Islander	5,119 (0.80)	5,434 (0.81)	7,197 (1.06)	7,464 (1.07)	7,465 (1.05)	8,389 (1.14)	7,227 (0.96)	48,295 (1.0)
Native American	2,457 (0.38)	3,925 (0.59)	4,676 (0.69)	4,057 (0.58)	4,910 (0.69)	6,237 (0.85)	5,213 (0.69)	31,476 (0.6)
Other	12,180 (1.90)	10,742 (1.60)	12,756 (1.89)	16,832 (2.40)	20,650 (2.90)	15,433 (2.10)	16,583 (2.20)	105,177 (2.2)
Insurance Status								
Self-pay	64,280 (10.01)	67,279 (10.03)	68,181 (10.43)	60,632 (8.65)	72,160 (10.14)	76,225 (10.38)	71,585 (9.51)	480,341 (9.8)
Medicare	255,489 (39.80)	267,036 (39.81)	270,529 (39.85)	283,820 (40.50)	291,340 (40.95)	300,939 (40.96)	321,304 (42.68)	1,990,456 (40.7)
Medicaid	83,723 (13.04)	86,135 (12.84)	82,374 (12.13)	84,877 (12.11)	93,423 (12.12)	102,481 (13.94)	102,649 (13.64)	635,663 (13.0)
Private insurance	201,080 (31.33)	203,042 (30.23)	213,567 (31.46)	229,545 (32.75)	212,401 (29.86)	211,529 (28.79)	215,050 (28.57)	1,486,214 (30.4)
No charge	7,962 (1.24)	8,876 (1.32)	6,969 (1.03)	8,178 (1.17)	7,787 (1.09)	7,577 (1.03)	7,973 (1.06)	55,323 (1.1)
Other	28,275 (4.41)	37,248 (5.55)	35,833 (5.28)	32,220 (4.60)	32,335 (4.55)	33,499 (4.56)	2,391 (0.32)	201,801 (4.12)
Discharge Disposition								
Routine	341,709 (53.24)	360,433 (53.73)	367,531 (54.14)	373,517 (53.30)	391,395 (55.02)	397,904 (54.16)	400,587 (53.22)	2,633,078 (53.8)
Short-term hospital	7,726 (1.20)	7,637 (1.14)	7,636 (1.12)	7,247 (1.03)	8,285 (1.16)	8,389 (1.14)	8,819 (1.17)	55,739 (1.1)
Skilled nursing facility	48,584 (7.57)	53,041 (7.91)	52,179 (7.69)	54,738 (7.81)	60,323 (8.48)	63,574 (8.65)	66,398 (8.82)	398,836 (8.2)
Intermediate care facility	3,856 (0.60)	4,364 (0.65)	4,068 (0.60)	4,738 (0.68)	4,579 (0.64)	5,251 (0.71)	5,190 (0.69)	32,045 (0.7)
Home health care	103,052 (16.06)	116,035 (17.30)	114,914 (16.93)	124,383 (17.75)	127,589 (17.94)	137,640 (18.74)	138,949 (18.46)	862,561 (17.6)
Against medical advice	9,523 (1.48)	10,932 (1.63)	10,194 (1.50)	9,502 (1.36)	10,774 (1.51)	11,765 (1.60)	11,870 (1.58)	74,560 (1.5)
Died	3,117 (0.49)	3,135 (0.47)	3,077 (0.45)	3,375 (0.48)	3,209 (0.45)	3,149 (0.43)	3,078 (0.41)	22,140 (0.5)
Other	124,295 (19.36)	115,242 (17.18)	119,278 (17.57)	123,303 (17.59)	105,237 (14.79)	106,991 (14.56)	117,881 (15.66)	812,227 (16.6)
Infection Type								
Other cellulitis and abscess	468,533 (73.00)	490,462 (73.11)	498,343 (73.41)	510,362 (72.83)	524,213 (73.69)	543,772 (74.02)	557,526 (74.06)	3,593,211 (73.46)
Postoperative wound infection	137,751 (21.46)	143,306 (21.36)	144,966 (21.35)	155,913 (22.25)	152,826 (21.48)	155,845 (21.21)	158,308 (21.03)	1,048,915 (21.45)
Cellulitis and abscess of fingers and toes	28,580 (4.45)	29,376 (4.38)	27,787 (4.09)	27,717 (3.96)	27,792 (3.91)	28,158 (3.83)	360,110 (4.00)	199,520 (4.08)
Other**	6,999 (1.08)	7,674 (1.14)	7,780 (1.14)	6,811 (0.98)	6,561 (0.92)	6,889 (0.94)	6,826 (0.96)	49,541 (1.02)
Hospital Region								
Northeast	128,929 (20.09)	138,493 (20.65)	141,487 (20.84)	145,384 (20.75)	148,953 20.94)	155,516 (21.17)	460,885 (21.37)	1,019,647 (20.9)
Midwest	141,412 (22.04)	151,838 (22.64)	151,927 (22.38)	156,106 (22.28)	164,547 (23.13)	167,645 (22.82)	170,873 (22.70)	1,104,348 (22.6)
South	253,939 (39.57)	261,603 (39.00)	265,645 (39.13)	276,150 (39.41)	275,532 (38.73)	282,269 (38.42)	292,204 (38.81)	1,907,344 (39.0)
West	117,454 (18.30)	118,796 (17.71)	119,809 (17.65)	123,157 (17.57)	122,403 (17.21)	129,345 (17.60)	128,890 (17.12)	859,855 (17.6)
Hospital Location								
Urban	548,876 (85.53)	570,881 (85.11)	582,750 (85.84)	601,566 (85.84)	608,981 (84.90)	627,281 (85.37)	640,677 (85.10)	4,76,011 (85.4)
Rural	88,980 (13.87)	94,924 (14.15)	93,918 (13.83)	98,428 (14.05)	96,405 (13.55)	99,723 (13.57)	103,914 (13.80)	676,293 (13.8)
Hospital Bed Size^†^								
Small	91,916 (14.32)	98,194 (14.64)	96,042 (14.15)	101,421 (14.47)	105,008 (14.76)	110,957 (15.10)	110,334 (14.66)	713,872 (14.6)
Medium	165,320 (25.76)	170,275 (25.39)	180,868 (26.64)	176,176 (25.14)	182,339 (25.63)	182,260 (24.80)	189,998 (25.24)	1,247,238 (25.5)
Large	380,619 (59.31)	397,335 (59.24)	399,757 (58.86)	422,398 (60.27)	413,038 (58.06)	433,787 (59.06)	444,260 (59.01)	2,891,195 (59.1)
Hospital Teaching Status								
Non-teaching	360,778 (56.23)	364,797 (54.39)	369,135 (54.38)	392,894 (56.06)	391,929 (55.09)	405,554 (55.19)	418,485 (55.59)	2,703,572 (55.3)
Teaching	277,078 (43.18)	301,008 (44.88)	307,533 (45.30)	307,101 (43.82)	308,456 (43.36)	321,450 (43.75)	326,107 (43.32)	2,148,732 (43.9)
Hospital Ownership								
Government, nonfederal	92,632 (14.43)	95,839 (14.29)	96,513 (14.22)	99,402 (14.18)	97,965 (13.77)	111,365 (15.16)	83,639 (11.11)	677,356 (13.9)
Private, non-profit	454,695 (70.85)	477,045 (71.12)	471,840 (69.50)	503,542 (71.85)	499,048 (70.15)	516,317 (70.27)	548,455 (72.85)	3,470,942 (71.0)
Private, invest-own	90,448 (14.09)	88,010 (13.12)	100,271 (14.77)	97,051 (13.85)	103,373 (14.53)	99,322 (13.52)	112,497 (14.94)	690,973 (14.1)

* Data presented as number of patients (%);

** Other infections include the following: other local infections of skin and subcutaneous tissues; posttraumatic wound infection, not elsewhere classified; and erysipelas; SSSI, skin and soft tissue infection;

^†^ The HCUP NIS bed-size categories vary based on geographic location (ie, northeast, midwest, south, and west), setting (ie, urban, rural), and teaching status; HCUP, Healthcare Cost and Utilization Project; NIS, National Inpatient Sample; SSSI, skin and skin structure infections

A large proportion of hospital admissions data for SSSIs were contributed by hospitals that were large in size (greater than 175 to 450 beds; 59.1%), from the South (39.0%), in an urban location (85.4%), privately (non-profit) owned (71.0%), and non-teaching (55.3%).

### SSSI-related Resource Utilization

**Fig 2:**
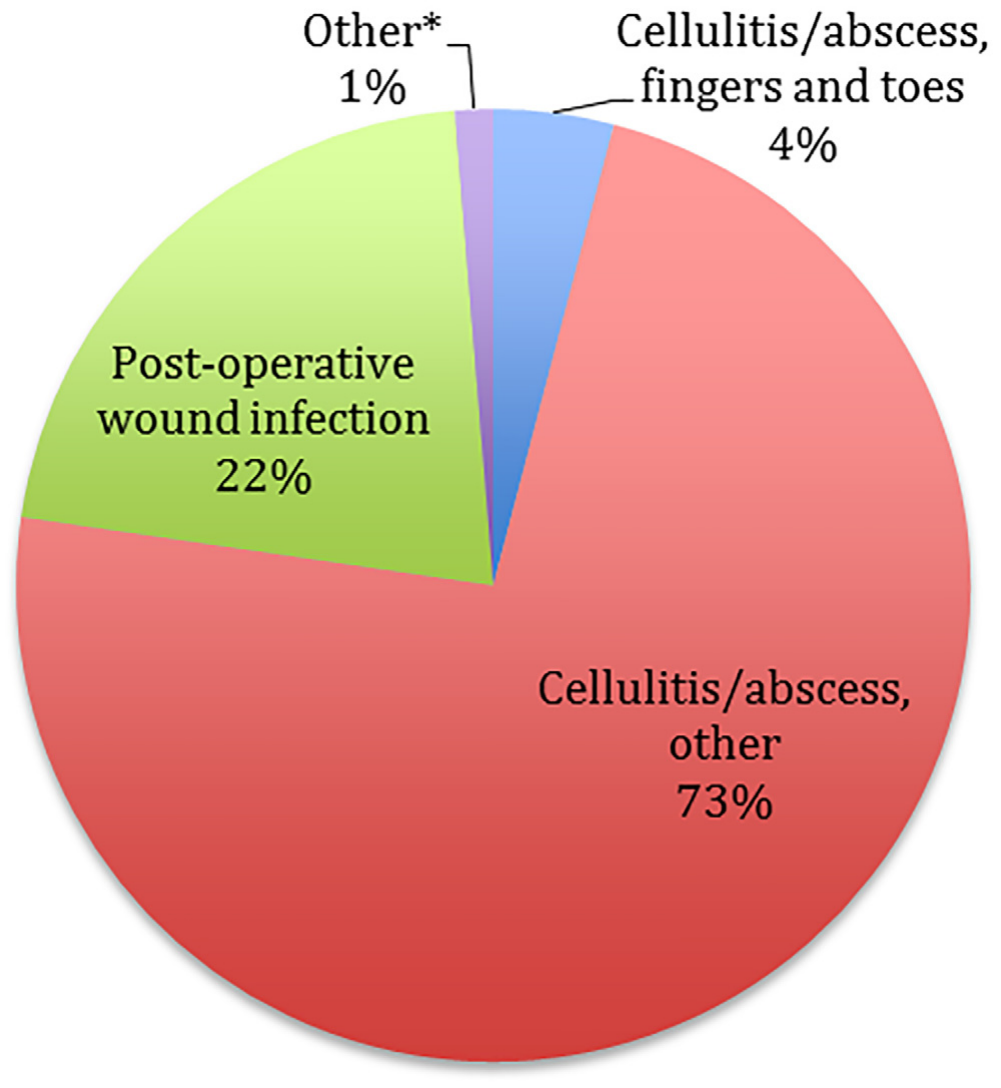
Hospital admission and mortality rates for SSSIs, years 2005–2011. *P ≤ .001 for trend; **P = .174 for trend; SSSI, skin and skin structure infection.

Admissions for primary SSSI infections significantly varied over the study period (*P* < .001) ranging from a low of 1.6% in the year 2005 to a high of 2.0% in the year 2011. The SSSI mortality rate did not differ over the assessed time period (*P* = .174 for trend; Fig 2). Mean hospital LOS reduced significantly (*P* < .001) during the study from 5.4 days in the year 2005 to 5.0 days in the year 2011; total inpatient costs did not differ (Table 3; *P* = .84 for trend).

**Table 3:** Mean (SD) length of stay and total costs for adult inpatients with a primary SSSI diagnosis.

Resource	Year						
	2005	2006	2007	2008	2009	2010	2011
Length of Stay, d*	5.40 (6.04)	5.39 (6.15)	5.25 (5.77)	5.24 (5.93)	5.16 (5.98)	5.11 (5.61)	4.95 (5.45)
Total Cost, $^†^	10,376.44 (16,841.62)	10,160.43 (15,547.65)	10,121.58 (16,398.00)	10,734.98 (18,019.29)	10,144.95 (16,163.89)	10,360.06 (15,909.36)	9,895.31 (15,393.92)

* Statistically significant change in mean length of stay (days) over the study period, *P* < .001;

^†^ 2012 US dollars; SD, standard deviation; SSSI, skin and skin structure infection

Patients with postoperative wound infections had the longest hospital stays (LS adjusted mean, 5.81 days; 95% confidence interval [CI], 5.80–5.83) and highest total costs (LS adjusted mean, $9388; 95% CI, $9366-$9410) after adjustment for patient demographics, hospital characteristics and APR DRG SOI. Hospital costs for inpatient SSSI admissions with a LOS of 3 to 6 days (LS adjusted mean, $8365; 95% CI, $8350-$8380) were 75% more expensive than those for hospital admissions of less than 3 days (adjusted mean, $4820; 95% CI: $4813-$4828).

### Multivariate Analyses

Numerous variables were determined to be significantly associated with mortality and total hospital costs in multivariate analyses (Table 4). Year of hospital admission was strongly associated with mortality, with chances of mortality significantly reducing 2007 onwards as compared to the year 2005. For instance, odds of mortality were significantly lower for hospital admissions in the year 2011 compared to 2005 (OR 0.42; 95% CI 0.37–0.48). Infection type, APR-DRG SOI level, and LOS were strongly associated with hospital costs after adjustment for other variables. Patients with postoperative wound infections had 30% greater hospital costs compared to patients with other cellulitis.

**Table 4:** Impact of patient and hospital characteristics on mortality and costs.

	Mortality			Total Costs		
Characteristics	Odds Ratio (95% CI)	*Χ* ^2^	*P*-Value	Exp Estimate (95% CI)	*Χ* ^2^	*P*-Value
Age, y						
18–44 (Reference)						
45–64	2.0 (1.7–2.3)	78.8	<.001	0.981 (0.979–0.984)	191.4	<.001
≥65	2.1 (1.8–2.5)	82.1	<.001	0.902 (0.898–0.905)	3057.4	<.001
Sex						
Female (Reference)						
Male	0.942 (0.888–0.998)	4.1	.04	1.022 (1.020–1.024)	418.7	<.001
Race						
White (Reference)						
Black	0.92 (0.82–1.04)	1.8	.18	1.07 (1.06–1.07)	1185.5	<.001
Hispanic/Latino	0.81 (0.71–0.92)	10.3	.001	1.06 (1.05–1.06)	715.1	<.001
Asian/Pacific Islander	1.0 (0.78–1.30)	0.0	.93	1.03 (1.02–1.05)	37.0	<.001
Native American	0.93 (0.60–1.44)	0.1	.74	1.00 (0.99–1.01)	0	.96
Other	0.87 (0.68–1.10)	1.4	.23	1.08 (1.07–1.09)	412.3	<.001
Admission Date, y						
2005 (Reference)						
2006	0.92 (0.82–1.04)	1.6	.20	0.995 (0.991–0.999)	5.7	.02
2007	0.84 (0.74–0.95)	7.9	.005	0.999 (0.995–1.003)	0.4	.53
2008	0.69 (0.61–0.78)	35.2	<.001	1.034 (1.029–1.038)	270.9	<.001
2009	0.54 (0.48–0.61)	97.2	<.001	0.994 (0.990–0.998)	8.4	.004
2010	0.47 (0.41–0.53)	141.0	<.001	1.017 (1.013–1.021)	69.2	<.001
2011	0.42 (0.37–0.48)	169.6	<.001	0.992 (0.988–0.996)	14.7	<.001
Insurance Status						
Medicare (Reference)						
Medicaid	1.12 (0.98–1.28)	2.9	0.09	1.028 (1.024–1.032)	186.3	<.001
Private	0.91 (0.82–1.00)	3.6	0.06	1.018 (1.015–1.022)	123.2	<.001
Self-pay	1.18 (0.95–1.45)	2.3	0.13	1.049 (1.044–1.054)	443.5	<.001
No charge	1.00 (0.62–1.61)	0	0.997	1.01 (1.00–1.02)	6.0	.01
Other	0.92 (0.73–1.18)	0.4	0.52	1.020 (1.015–1.026)	51.8	<.001
Hospital Region						
South (Reference)						
Northeast	1.15 (1.04–1.28)	7.2	.007	1.283 (1.279–1.287)	26209.9	<.001
Midwest	0.84 (0.76–0.93)	10.6	.001	1.070 (1.067–1.073)	1961.9	<.001
West	0.95 (0.86–1.04)	1.3	.26	1.855 (1.849–1.862)	116486.0	<.001
Hospital Location						
Urban (Reference)						
Rural	1.27 (1.14–1.41)	18.9	<.001	0.991 (0.987–0.994)	33.0	<.001
Type of Infection						
Other cellulitis (Reference)						
Cellulitis/abscess of toe and fingers	0.60 (0.46–0.79)	13.2	<.001	1.07 (1.06–1.08)	610.7	<.001
Postoperative wound infection	1.10 (1.03–1.18)	8.3	.004	1.300 (1.296–1.303)	36924.8	<.001
Other infections	1.33 (0.97–1.81)	3.1	.08	1.09 (1.08–1.12)	288.2	<.001
Hospital Bed Size*						
Large (Reference)						
Medium	1.01 (0.93–1.09)	0.0	.88	1.019 (1.017–1.022)	231.6	<.001
Small	1.21 (1.09–1.34)	13.5	<.001	1.096 (1.093–1.1)	3497.3	<.001
Hospital Ownership						
Private, nonprofit (Reference)						
Private, investor-owned	1.26 (1.13–1.39)	18.7	<.001	0.972 (0.969–0.975)	302.9	<.001
Government, nonfederal, public	1.29 (1.17–1.43)	25.6	<.001	1.043 (1.040–1.046)	702.4	<.001
Hospital Teaching Status						
Non-teaching (Reference)						
Teaching	1.01 (0.93–0.10)	0.1	0.76	1.013 (1.011–1.015)	119.48	<.001
APR-DRG ROM Level^†^				APR-DRG SOI Level^†^		
1 (Reference)						
2	7.58 (6.19–9.28)	384.4	<.001	1.119 (1.116–1.122)	7112.6	<.001
3	58.21 (47.91–70.71)	1674.93	<.001	1.331 (1.327–1.336)	28881.8	<.001
4	624.39 (514.12–758.31)	4215.2	<.001	2.39 (2.37–2.40)	71573.7	<.001
Discharge Type						
Routine (Reference)						
Short-term hospital				1.14 (1.13–1.15)	677.9	<.001
Skilled nursing facility				1.128 (1.123–1.132)	3151.5	<.001
Intermediate care facility				1.02 (1.01–1.04)	11.2	<.001
Home health care				1.112 (1.109–1.115)	4951.0	<.001
AMA				0.88 (0.87–0.89)	878.06	<.001
Died				1.61 (1.59–1.64)	3522.54	<.001
Length of Stay, d						
0–3 (Reference)						
3–6				1.735 (1.731–1.740)	199735	<.001
> 6				3.62 (3.61–3.63)	675141	<.001

AMA, against medical advice; APR-DRG, all patient refined diagnosis related group; CI, confidence interval; d, day; Exp Estimate, exponentiation of the beta-estimate; ROM, risk of mortality; SOI, severity of illness

## Discussion

Hospital admissions for SSSIs continue to account for a growing percentage of hospitalizations in the United States. Admissions for primary SSSI infections significantly varied over the study period (from 1.6% to 2.0%), which could translate into about 650 to 800 hospitalizations annually at a large teaching medical center (assuming approximately 40,000 admissions per year). We found an estimated 17% increase in the total number of adult hospitalizations to US acute-care, non-federally funded hospitals from the year 2005 through the year 2011 for SSSIs. It is possible that rates of hospitalization are increasing as a result of multi-drug resistance (MDR) increase globally. MDR pathogens, such as MRSA, are associated with increased rates of treatment failure. In addition, the vast majority of SSSI hospital admissions were for patients with comorbidities; diabetes was the most common comorbidity. The number of SSSI hospital admissions for patients with no comorbidities decreased 37% from the year 2005 through the year 2011, which might also explain the increase in hospitalization rates during the study period. In addition, age of the cohort increased over time, which might also explain the increased need for hospitalization in the latter years of the study.

Although mean hospital LOS decreased from 5.4 days in the year 2005 to 5.0 days in the year 2011, mean hospital costs remained stable. As expected, hospital costs increased with increasing LOS; costs were 3-times higher for admissions lasting more than 3 days than those lasting less than 3 days. Factors that might explain why LOS decreased over the study period include changes in SSSI treatment regimens, increased utilization of clinical guidelines or pathways, improved recognition of resistant pathogens such as MRSA, strong healthcare reform environment to manage and treat patients more efficiently, increased administration of appropriate empiric antibiotic therapy, availability of oral antibiotics that can allow early discharge and switch to oral formulations, and shortened hospital stays in general for patients.

Mean costs per admission were found to be stable despite the reduction in LOS, which could be explained by increased use of new antimicrobials available for treatment of SSSI, that are more expensive than generic vancomycin or cephalosporins (daptomycin, telavancin, linezolid etc).

The implications of our findings suggest that SSSI will continue to be an area where hospital policy makers focus. The reductions in LOS findings indicate that many patients may be continuing treatment outside of the hospital (either outpatient infusion centers or new oral therapies). With Affordable Care Act legislation, there will continue to be strong incentives to shift care to outpatient settings. The treatment paradigm will be shifted as the external policies continue to challenge health care decision-makers to find better ways to manage patient efficiently in non-hospital settings.

Results from our study are similar to and expand upon those reported by Edelsberg et al. and Suaya et al.[8, 9] Edelsberg identified all hospital admissions in the HCUP NIS database with a principle diagnosis of SSSI for the years 2000 through 2004 regardless of age.[8] Edelsberg used a broader definition for SSSI that included more ICD-9 codes compared to this study. Results from this study showed a 29% increase in SSSI hospitalizations from the year 2000 through the year 2004.

Suaya et al. used the HCUP NIS database to quantify the incidence and associated costs for *S aureus* SSSIs from the year 2001 to the year 2009.[9] All patients with a *S aureus* infection were identified using ICD-9-CM codes. Patients with a concomitant SSSI diagnoses were then identified using additional ICD-9-CM codes (most of which were not included in our study). Results from this study showed a 123% increase in *S aureus* SSSI in the United States from 160,881 in the year 2001 to 358,212 in the year 2009. Additionally, the share of *S aureus* hospitalization represented by SSSIs increased from 39% in the year 2001 to 51% in the year 2009. Although adults 75 years of age or older had the smallest increase in *S aureus* SSSI incidence from the year 2001 to 2009, they had the overall highest *S aureus* SSSI hospitalization incidence across all studied years.

In addition to trends in *S aureus* SSSI incidence, Suaya also evaluated healthcare resource utilization for this patient population. From the year 2001 to 2009 *S aureus* SSSI hospitalization costs increased 26% implying an increase in national costs from $3.36 to $4.22 billion ($US 2010). The authors postulated that national costs did not increase more due to the lower age of those hospitalized and an overall decrease in hospital LOS.

Although our results continue to show an increase in SSSI hospitalizations, data from the last 5 years suggest that this increase may be slowing. From the year 2007 through the year 2011, we found only an 11% increase in SSSI hospitalizations with the greatest increase occurring in patients 65 years of age or older. In contrast, Edelsberg reported that the greatest increase in SSSI admissions was for patients less than 65 years of age compared to those 65 to 100 years of age and Suaya reported the greatest increase in *S aureus* SSSI hospitalizations in children 0 to 17 years of age (318% increase) and adults aged 18 to 44 years (173% increase). A potential explanation for this difference in infection rates for these age ranges may be the differences in ICD-9-CM codes utilized in these studies or the possibility that more of the younger patients are now receiving only outpatient therapy for SSSIs.

Edelsberg et al. also reported greater increases in SSSI admissions for patients seen in urban rather than rural hospitals, and those admitted for treatment of superficial infections such as cellulitis and abscess compared to those with deeper or healthcare-associated infections such as postoperative wound or vascular device infections. We did not find a difference in the increase in SSSI admissions between urban and rural hospitals or in SSSI admissions for cellulitis and abscess than other postoperative wound infections. In general, the relative proportions of different infection types remained stable throughout the study although there was a slight numerical increase in the percentage of patients diagnosed with cellulitis or abscess from the year 2005 (73%) through the year 2011 (74.1%).

Hospital LOS decreased in the study by Suaya et al. from 9.91 days in the year 2001 to 7.26 days in the year 2009, but it was still longer than the 5.4 to 5.0 day LOS we reported. This difference in LOS may reflect differences in study populations in that our study enrolled only patients with a primary SSSI whereas Suaya included patients with a primary or secondary *S aureus* SSSI diagnosis. Similarly, the cost of hospitalization was higher in the study by Suaya than our study ranging from $21,287 in the year 2001 to $11,622 in the year 2009. The higher costs found by Suaya may reflect the use of associated cost estimates that include the costs of concomitant comorbidity treatments. In both studies however, hospital costs decreased over the study period potentially reflecting the decrease in hospital LOS seen in both studies.

Results from our multivariate analyses identified characteristics that increased or decreased mortality risk as well as healthcare resource utilization in adult patients hospitalized in the United States with a primary SSSI diagnosis. We were not surprised by many of these results such as increasing mortality risk with increasing APR-DRG ROM level and increasing costs with increasing APR-DRG SOI level. One covariate strongly associated with mortality was year of hospital admission. Although the HCUP NIS database does not contain information on drugs administered or organisms cultured, one possible explanation for this decrease in mortality seen over time is the earlier administration of antibiotics with MRSA activity. As healthcare providers became more aware of the increase in MRSA SSSIs, including those caused by CA-MRSA, the use of empiric therapy targeted at this organism may have increased, decreasing the time to appropriate antibiotic therapy administration and ultimately mortality. Results from a study conducted in patients hospitalized for SSSI treatment suggest a 3-fold increase in mortality in patients who failed initial antibiotic therapy (OR, 2.91; 95% CI: 2.34–3.62).[11]

The utilization of the HCUP NIS dataset contributes to both the strengths and limitations of this study. The HCUP NIS dataset is a large database representing approximately 20% of US hospital admissions; selection bias is minimized through the use of weighted numbers supplied through the HCUP. We aimed to decrease the influence of comorbidities on healthcare resource utilization estimates by limiting our study population to those with a primary SSSI diagnosis. Additionally, we used ICD-9-CM codes specific to those infections identified as ABSSSIs in the US FDA guidance document.[1]

Results from this analysis should be evaluated keeping the following limitations in mind. First, because of the large amount of data contained in the NIS database, small differences can be statistically significant. Second, the HCUP database tracks hospital admissions and not patients meaning an individual may be responsible for multiple admissions. Third, the HCUP dataset does not include information on specific organisms or medication administration records therefore we were not able to determine why SSSI admissions increased, only that they increased. Fourth, our results are limited by problems inherent to database analyses such as medical coding errors, missing patient record data, and misclassifications. Lastly, the authors provided rationale for the key findings from this study, such as increasing ABSSSI hospital admissions, decreasing LOS and stable costs. However, this rationale is speculative and is based on the authors' best judgement and experience and on previously published data. Future research is needed to further explore and understand these findings.

## Conclusion

Our study results show that the number of hospital admissions for adult patients with a SSSI primary diagnosis continues to increase in the United States. Even with the cost per case remaining stable, this increase in hospital admissions contributes to both the clinical and economic burden that SSSI patients add to the US healthcare system. The decreasing hospital inpatient LOS and mortality rate may be indicative of improved management and therapy of patients. Future research should identify potential opportunities to shift SSSI management from inpatient to outpatient treatment settings. The recent approval of new long-acting antibiotics (eg lipoglycopeptides, such as dalbavancin, oritavancin) offer unique therapeutic options that can facilitate outpatient management for some patients with SSSI.
